# Perceptions and Attitudes Regarding Hematopoietic Stem Cell Donation Among Blood Donors in Riyadh, Saudi Arabia

**DOI:** 10.7759/cureus.51406

**Published:** 2023-12-31

**Authors:** Faris Alsalamah, Mohammed K Alageel, Rakan Alzahrani, Yazeed Alowairdhi, Nasser Alkahtani, Emad Masuadi, Dunia Jawdat

**Affiliations:** 1 Department of Medicine, King Abdulaziz Medical City, Riyadh, SAU; 2 Department of Orthopedics, Ad Diriyah Hospital, Riyadh, SAU; 3 Department of Family Medicine, Family Medicine Academy, Eastern Health Cluster, Dammam, SAU; 4 Department of Dermatology, King Abdulaziz Medical City, Riyadh, SAU; 5 Department of Emergency Medicine, King Abdulaziz Medical City, Riyadh, SAU; 6 Department of Medicine, King Saud Bin Abdulaziz University for Health Sciences, Riyadh, SAU; 7 Department of Allergy and Immunology, Saudi Stem Cell Donor Registry, King Abdullah International Medical Research Center, Riyadh, SAU

**Keywords:** stem cell donor, voluntary blood donation, blood donors, donation, stem cell, attitude, perception

## Abstract

Introduction

In recent years, there has been a growing trend toward using stem cell transplantation for therapeutic purposes, making a positive impact in the medical field. Access to a compatible and willing donor is essential for those therapeutic purposes, yet the current number of registered donors remains inadequate. The present study aimed to investigate the attitude and perception of stem cell donation among blood donors in Saudi Arabia while also exploring their knowledge of hematopoietic stem cells, willingness towards donation, and fear of complications after stem cell donation.

Methods

A cross-sectional study was implemented to investigate the perception and attitude toward stem cell donation among blood donors in Riyadh, Saudi Arabia, through a validated self-administered questionnaire. The questionnaire comprised 35 questions divided into five sections, namely, demographics, knowledge, attitude, willingness, and fear of stem cell donation.

Results

The survey was distributed to 400 subjects. Out of the 400 respondents, 98.8% (n=395) were male, and 90.8% (n=363) were Saudi nationals. The majority had a high school level of education (n=259, 64.75%). Only 10.8% (n=43) of the participants were knowledgeable about stem cells. Knowledge of stem cells was highest among females aged 40-49 years, participants knowledgeable of platelet donation, and participants who donated blood more than 10 times (p-value <0.05). Participants with a bachelor's or master's degree had significantly more fear of stem cell donation complications, with a p-value of 0.003. The attitude toward stem cell donation was highly positive. Most participants strongly agreed to donate stem cells to a family member or anyone in need, 94.5% (n=378) and 62% (n=248), respectively.

Conclusion

Knowledge about stem cell donation among blood donors was scarce, while their willingness to donate after conversing was high. We highly recommend the initiation and establishment of educational programs to increase the knowledge of the public and, specifically, blood donors.

## Introduction

Stem cells serve as the fundamental components of every living organism. Although present in all multicellular organisms, these cells are most active during the initial stages of human development and can still be found in certain areas of the body during adulthood [1]. The two defining traits of stem cells include their ability to constantly renew themselves and their capacity to differentiate into specific types of specialized adult cells [2]. Hematopoietic stem cell transplantation is beneficial in the medical field nowadays, and the procedure's success depends significantly upon the matching of human leukocyte antigen (HLA) between the donor and recipient [3]. The probability of finding a matched donor varies among different countries. Recent data showed that the probability of finding a matched family donor in Jordan and Pakistan was as high as 65% and 70%, respectively [4,5]. Locally, a study reported the probability of finding a sibling matching the patient's HLA as 60%. However, the study also reported that 57% of children from birth to five years and 32% of adults who need stem cell transplantation do not find a matching family member [6]. Consequently, many countries, including Saudi Arabia, have established registries of stem cell donors to increase the probability of matching patients in need of stem cell donation with those willing to donate.

The Saudi Stem Cell Donor Register (SSCDR) was established in April 2011. Currently, the total count of registered Saudi stem cell donors is 79,742, with an average of 6,645 new registrations per year [7]. This number accounts for only 0.25% of the total population of Saudi Arabia, which is estimated to be more than 32 million [8]. When comparing the number of currently registered stem cell donors to the annual number of Saudi blood donors, which was 342,460 in 2020 [9], an enormous difference between the two can be noticed. Also, a recent study reported that 58% of Saudis agree and frequently donate their blood [10]. This also supports the observation that the number of registered stem cell donors is relatively low compared to the number of blood donors. Moreover, considering international figures, this phenomenon seems widespread globally. For instance, in the United States, only 2% of its population is on the national stem cell registry [11]. The case is the same for the United Kingdom, as it was estimated that only 2% of their population is registered as stem cell donors. However, the proportion is relatively higher, yet low, in other countries; for instance, 13% in Cyprus and 9% in Germany [12].

The present study aimed to investigate the attitude and perception of stem cell donation among blood donors in Saudi Arabia while also exploring their knowledge of stem cells, willingness towards donation, and fear of stem cell donation and its complications. Due to the lack of sufficient evidence on the perception and attitude of stem cell donation among blood donors in Riyadh, Saudi Arabia, our study provides valuable insight into the above-mentioned parameters in blood donors.

## Materials and methods

Study design and settings

A validated, self-administered questionnaire that examined several factors contributing to the participant's decision to register as a stem cell donor was administered. These factors included knowledge and attitude toward stem cell donation, as well as willingness and fear of doing so. The study was conducted at the King Abdulaziz Medical City (KAMC) Blood Donation Center in Riyadh, Saudi Arabia. This center is one of Riyadh's largest blood donation centers, receiving more than 36,000 blood donors annually.

Study subjects

The study included healthy blood donors older than 18 years and younger than 49 years who donated blood at the KAMC Blood Donation Center during the data collection period. The exclusion criteria were blood donors who had previously donated stem cells.

Sample size and technique

The sample size was calculated at the 95% confidence level. The expected response for the outcome variable, a positive attitude towards stem cell donation, was kept at 50%. The minimum required sample size for a margin of accuracy of 5% was estimated to be 381. A non-probability convenience sampling approach was utilized. We selected all individuals who donated blood at the KAMC Blood Donation Center at the time of data collection and met inclusion and exclusion criteria.

Data collection

Blood donors who met the inclusion criteria were asked to fill out an informed consent form and a validated questionnaire of 35 closed-ended questions. The questionnaire included six questions to identify participants' demographics, 19 to assess their knowledge of stem cell donation, four to evaluate their attitude towards stem cell donation, two to determine their willingness to donate, three to evaluate their fear, and finally, one question to identify their preferred method of donation.

Statistical analysis

Data were collected, reviewed, and entered in Microsoft Excel, then analyzed using the IBM Statistical Package for Social Sciences software version 26 (IBM Corp., Armonk, NY). The descriptive statistics were presented as frequency and percentage for the categorical data. The numerical variables were presented as means and standard deviations. The Chi-square test was used to analyze the data, in which both the predictor and outcome were categorical. A p-value < 0.05 was considered significant for all the statistical tests.

Ethical Approval

The study received approval from the Institutional Review Board (IRB) committee at King Abdullah International Medical Research Center (KAIMRC), Riyadh, Saudi Arabia, with the reference number SP19/370/R.

## Results

The survey was distributed to 400 subjects, with a 97% response rate. The demographics section included age, gender, level of education, nationality, blood donation regularity, and knowledge regarding platelet donation and stem cell donation. Of all participants, the majority were male (n=395, 98.8%), Saudi nationals (n=363, 90.8%), and of high school level of education (n=259, 64.75%). The percentage of knowledgeable participants was low (n=43, 10.8%) (Table 1).

**Table 1:** Demographic characteristics of the population studied

Variable	Category	Total = 400, n (%)
Gender	Male	395 (98.8%)
	Female	5 (1.2%)
Age	18–29	230 (57.5%)
	30–39	131 (32.8%)
	40–49	39 (9.7%)
Level of education	Less than high school	23 (5.75%)
	High school degree	259 (64.8%)
	Bachelor's degree	103 (25.8%)
	Master’s degree	13 (3.2%)
	Doctorate	2 (0.5%)
Nationality	Saudi	363 (90.8%)
	Non-Saudi	37 (9.75%)
Frequency of blood donation	First time	52 (13%)
	Less than 5 times	136 (34%)
	5-10 times	113 (28.2%)
	More than 10 times	99 (24.8%)
Knowledge of platelet donation	Knowledgeable	215 (53.8%)
	Non-knowledgeable	185 (46.2%)
Knowledge of stem cell donation	Knowledgeable	43 (10.8%)
	Non-knowledgeable	357 (89.2%)

The age group of 40-49 years had a higher level of knowledge than other age groups, with a significant p-value of 0.029. Additionally, females had a higher level of knowledge than males, with a significant p-value of 0.010. When comparing the level of education with the level of knowledge, participants with a doctorate, master's, or bachelor's degree had higher knowledge than other groups with a p-value less than 0.05. Participants who were knowledgeable about platelet donation had higher knowledge about stem cells, with a significant p-value of 0.001. According to the frequencies of blood donation, people who donated more than 10 times had higher knowledge with a significant p-value of less than 0.05 (Table 2).

**Table 2:** Association between dependent variables and knowledge of stem cell donation

Dependent variables	Category	Knowledgeable, n (%)	Non-knowledgeable, n (%)	p-value
Age (in years)	18-29	17 (7.4%)	213 (92.6%)	0.029
	30-39	19 (14.5%)	112 (85.5%)	
	40-49	7 (17.9%)	32 (82.1)	
Gender	Male	40 (10.1%)	355 (89.9%)	0.010
	Female	3 (60%)	2 (40%)	
Level of education	Less than high school	0 (0%)	23 (100%)	0.000
	High school degree	14 (5.4%)	245 (94.6%)	
	Bachelor’s degree	22 (21.4%)	81 (78.6%)	
	Master’s degree	5 (38.5%)	8 (61.5%)	
	Doctorate	2 (100%)	0 (0%)	
Frequency of blood donation	First time	6 (11.5%)	46 (88.5%)	0.000
	Less than 5 times	7 (5.1%)	129 (94.9%)	
	5-10 times	6 (5.3%)	107 (94.7%)	
	More than 10 times	24 (24.2%)	75 (75.8%)	
Knowledge of platelet donation	Knowledgeable	34 (15.8%)	181 (84.2%)	0.001
	Non-knowledgeable	9 (4.9%)	176 (95.1%)	

Table 3 summarizes the participants' fear of stem cell donation complications correlated with their level of education.

**Table 3:** Association between the participants' level of education and fear of stem cell donation complications

Level of education	Fear of complications, n (%)				p-value
	None	Low	Moderate	High	
Less than high school	13 (56.5%)	8 (34.8%)	0 (0%)	2 (8.7%)	0.003
High school degree	191 (73.7%)	23 (8.9%)	23 (8.9%)	22 (8.5%)	
Bachelor’s degree	60 (58.2%)	13 (12.6%)	15 (14.6%)	15 (14.6%)	
Master’s degree	7 (53.8%)	1 (7.7%)	3 (23.1%)	2 (15.4%)	
Doctorate	1 (50%)	0 (0%)	1 (50%)	0 (0%)	

Most participants had no fear of stem cell complications (n=272, 68%). However, those with a master's or bachelor's degree had higher levels of fear. In contrast, those with a high school degree demonstrated lower levels of fear of stem cell donation and its complications, with a significant p-value of 0.003.

Most blood donors preferred to donate stem cells through peripheral blood stem cell apheresis rather than bone marrow harvest (n=380, 95%). The fear level of blood donors about the stem cell donation method was higher for bone marrow harvest than for peripheral blood stem cell apheresis. Moreover, the knowledgeable participants' attitudes, willingness, and fear of stem cell donation showed no significant difference between age groups, gender, level of education, knowledge of platelet donation, and blood donation frequency (Tables 4-6).

**Table 4:** The participants' attitude towards stem cell donation

Statements and questions	Answers	Total = 43, n (%)
Stem cell transplantation should be applied widely.	Strongly agree	36 (83.7%)
	Agree	6 (14%)
	Neutral	0 (0%)
	Disagree	1 (2.3%)
	Strongly disagree	0 (0%)
There should be more awareness programs on stem cells.	Strongly agree	40 (93%)
	Agree	3 (7%)
	Neutral	0 (0%)
	Disagree	0 (0%)
	Strongly disagree	0 (0%)
If there is a campaign for a specific patient in my area, I would like to join.	Strongly agree	26 (60.5%)
	Agree	12 (27.9%)
	Neutral	2 (4.6%)
	Disagree	3 (7%)
	Strongly disagree	0 (0%)
I would be interested in developing my knowledge of stem cells.	Strongly agree	31 (72.1%)
	Agree	8 (18.6 %)
	Neutral	3 (7%)
	Disagree	1 (2.3%)
	Strongly disagree	0 (0%)

**Table 5:** The participants' willingness towards stem cell donation

Statements and questions	Answers	Adequate Knowledge (n = 43), n (%)	Inadequate knowledge (n = 357), n (%)	Total = 400, n (%)
Would you donate stem cells if a matching family member is in need?	Strongly agree	42 (97.7%)	336 (94.1%)	378 (94.5%)
	Agree	1 (2.3%)	16 (4.5%)	17 (4.2%)
	Neutral	0 (0%)	4 (1.1%)	4 (1%)
	Disagree	0 (0%)	0 (0%)	0 (0%)
	Strongly disagree	0 (0%)	1 (0.3%)	1 (0.3%)
Would you donate stem cells if a matching person is in need?	Strongly agree	30 (69.8%)	218 (61.1%)	248 (62%)
	Agree	4 (9.3%)	74 (20.7%)	78 (19.5%)
	Neutral	9 (20.9%)	57 (16%)	66 (16.5%)
	Disagree	0 (0%)	6 (1.7%)	6 (1.5%)
	Strongly disagree	0 (0%)	2 (0.6%)	2 (0.5%)
Which method of donation would you prefer?	Via bloodstream	40 (93%)	340 (95.2%)	380 (95%)
	Via bone marrow	3 (7%)	17 (4.8%)	20 (5%)

**Table 6:** Fear of stem cell donation and its complications

Question	Answers	Adequate Knowledge (n = 43), n (%)	Inadequate knowledge (n = 357), n (%)	Total = 400, n (%)
Are you afraid of donating stem cells via the bloodstream?	None	34 (79.1%)	08 (86.3%)	342 (85.5%)
	Low	6 (14%)	24 (6.7%)	30 (7.5%)
	Moderate	3 (7%)	18 (5%)	21 (5.2%)
	High	0 (0%)	7 (2%)	7 (1.8%)
Are you afraid of donating stem cells via bone marrow?	None	15 (34.9%)	109 (30.5%)	124 (31%)
	Low	5 (11.6%)	59 (16.5%)	64 (16%)
	Moderate	13 (30.2%)	95 (26.6%)	108 (27%)
	High	10 (23.3%)	94 (26.3%)	104 (26%)
Do you have any fear of complications from stem cell donation?	None	26 (60.5%)	246 (68.9%)	272 (68%)
	Low	4 (9.3%)	41 (11.5%)	45 (11.2%)
	Moderate	8 (18.6%)	34 (9.5%)	42 (10.5%)
	High	5 (11.6%)	36 (10.1%)	41 (10.3%)

## Discussion

The present study explores blood donors' knowledge, attitude, willingness, and fear levels regarding stem cell donation. Blood donors' general knowledge of stem cell donation was deficient, as only 10.8% were knowledgeable, with a 56.3% (± of 22.3%) mean score for stem cell knowledge assessment. These findings demonstrate the need to increase blood donors' awareness of stem cell donation.

Knowledgeable participants were asked to choose their preferred method of stem cell donation out of the two main methods of stem cell donation, the first being bone marrow harvest and the second being peripheral blood stem cell apheresis, a unique type of leukapheresis designed to gather cells from the bloodstream for potential use in bone marrow transplants [13]. Knowledgeable donors' fear levels of stem cell donation correlated with their most preferred choice of donation method, peripheral blood stem cell apheresis. Only 7% had a moderate or high fear of donating stem cells through peripheral blood stem cell apheresis. On the other hand, 53.5% had moderate or high levels of fear when asked about the bone marrow harvest method. The distribution of fear levels between the two methods could explain the overwhelming tendency to choose peripheral blood stem cell apheresis as the preferred donation method (93%).

Few studies have explored the perception and attitude toward stem cell donation in the past [14-22]. These studies were aimed at either the general population [19,20,22], medical practitioners [14,18], medical students [15,16,18], or nursing students [17,20]. A recently published article reviewed whole-blood donors' willingness, motives, barriers, and interventions related to the donation of another substance of human origin, such as human organ donation [23]. Although knowledge assessment tools were not unified in past studies, knowledge of stem cells among the general population, healthcare practitioners, and students was reported to be low and moderate in many countries [14-19]. A study conducted in Jazan, Saudi Arabia, reported medical students' knowledge regarding donor eligibility and the donation process. Among all respondents, the average knowledge of donation eligibility and the donation process was 37.4% and 23.6%, respectively [14].

The outcomes of stem cell educational interventions for the public and healthcare practitioners have been assessed in two previous studies [19, 20]. Raising awareness about stem cell donation among healthcare providers and the general population is essential. It will ultimately increase enrollment in stem cell registries, which in turn will help advance the use of stem cells in medical applications for therapeutic and research purposes.

Since it was founded, the Saudi Stem Cell Donor Register (SSCDR) has been making great efforts to recruit new stem cell donors into the Saudi stem cell registry. Our findings suggest that targeting blood donors would be highly valuable, as the combination of low knowledge and a high willingness to donate presents a significant opportunity for educational interventions. Education can bridge the knowledge gap and harness the existing goodwill to bolster the ranks of stem cell donors. We urge a concerted effort to heighten public awareness, with a particular focus on the cohort of blood donors who have already demonstrated altruism in their readiness to donate blood. Additionally, by enhancing the understanding of stem cell donation among this group, we anticipate not only an expansion in the donor registry but also a reinforcement of the positive attitudes that underpin this critical form of medical donation. Ultimately, this approach will hopefully increase the annual Saudi stem cell registrations, leading to an increase in the number of patients who receive stem cell transplantation.

The cross-sectional study was appropriate to investigate the perception and attitude toward stem cell donation among blood donors and to investigate multiple dependent and independent variables. It provided the ability to select a sufficient sample of the population efficiently and effectively. On the other hand, the study has limitations due to its inability to investigate longitudinal changes in perception and attitude. Additionally, this study included blood donors in a single center; thus, it might not be fully representative of the general population.

## Conclusions

In conclusion, despite a low level of knowledge about stem cell donation among blood donors in Saudi Arabia, there is a remarkable willingness to contribute to this life-saving process. The findings point to a positive disposition towards stem cell donation among those who are informed, coupled with a generally low degree of apprehension regarding the donation process and its potential complications. Moreover, further studies are needed to consider our limitations and comprehensively assess how educational programs affect the attitudes of blood donors toward stem cell donation.

In light of our findings, we advocate for targeted strategies that focus on blood donors as a key demographic. This strategic approach could potentially maximize the efficacy of awareness campaigns and significantly increase the annual registration of stem cell donors. Such an increase is pivotal to ensuring a higher number of patients can benefit from stem cell transplantation, ultimately saving more lives and advancing medical science.
